# Molecular Cloning, Expression and Peroxidase Conjugation of *Staphylococcus aureus* Protein A

**DOI:** 10.15171/ijb.1267

**Published:** 2016-12

**Authors:** Masoud Reza Seyfi Abad Shapouri, Pezhman Mahmoodi, Dariush Gharibi, Masoud Ghorbanpoor, Sara Yaghoubi, Elham Rezaei, Mohammad Rashno, Neda Mehravar

**Affiliations:** ^1^Department of Pathobiology, Faculty of Veterinary Medicine, Shahid Chamran University of Ahvaz, Ahvaz, Iran; ^2^Department of Pathobiology, Faculty of Veterinary Science, Bu-Ali Sina University, Hamedan, Iran; ^3^Department of Immunology, School of Medicine, Ahvaz Jundishapur University of Medical Sciences, Ahvaz, Iran; ^4^Department of Biology, Faculty of Sciences, Shahid Chamran University of Ahvaz, Ahvaz, Iran

**Keywords:** Horseradish peroxidase, Staphylococcal protein A, SPA, *Staphylococcus aureus*

## Abstract

**Background:**

Staphylococcal protein A (SPA) is a cell wall component of *Staphylococcus aureus* that binds to different IgG subclasses of human and several animal species. This bacterial protein can be used as an antibody detector in various immunological assays or as an isolation reagent for the purification of antibody molecules via immuno-chromatography procedures.

**Objectives:**

Molecular cloning and expression of SPA followed by the purification and conjugation of the recombinant protein to peroxidase enzyme.

**Material and Methods:**

Encoding DNA fragment of SPA was amplified and inserted into a prokaryotic plasmid vector for the expression of recombinant SPA fused to a maltose binding protein (MBP). The recombinant protein was purified using amylose resin column chromatography and conjugated to horseradish peroxidase (HRP) enzyme. Finally, the reactivity of the recombinant SPA was examined against human IgG molecules in ELISA.

**Results:**

The results indicated that the recombinant peroxidase-conjugated SPA has a good recognition capacity for human IgG molecules and it was able to produce significant OD values after reacting with human IgG molecules at a concentration up to 0.06 μg.well^-1^.

**Conclusions:**

This recombinant protein can be very useful in all research laboratories and may decrease some of the expenses, *e.g.* those for preparing conjugated anti-antibodies.

## 1. Background

Detection of immunoglobulins is one of the most important concerns of researchers in the immunological processes. Generally, anti-antibody molecules are used for this purpose; however, it is a very expensive and time consuming procedure to produce such molecules. There are some natural proteins that bind to different immunoglobulins such as staphylococcal protein A (SPA), isolated from *Staphylococcus aureus*, protein G, isolated from group C or G streptococci, protein L, isolated from the anaerobic bacterium *Peptostreptococcus magnus*, and *Haemophilus somnus* immunoglobulin binding proteins (IgBPs) that consist of a group of high molecular weight proteins, which primarily bind to the bovine IgG2 (1-5). These proteins can obviously be proper substitutes for anti-antibody molecules. Among these, SPA is a well-known protein capable of adhering to different immunoglobulin molecules. This protein is a bacterial cell wall component produced by several strains of *Staphylococcus aureus,* although its homologs have been reported in other staphylococci, e.g. *Staphylococcus hyicus* (6). SPA is a single polypeptide chain protein that covalently bound to the cell wall peptidoglycan and contains little or no carbohydrate (1).

The interaction between protein A and antibodies has been studied in great detail and the binding is very well understood. Extensive hydrophobic interactions are evident with both the second and third constant regions of Fc domain of immunoglobulins (7). This protein contains five domains in tandem (E, D, A, B, and C) that each of which can interact with Fc or Fab regions of human immunoglobulins (8). Protein A also has the ability to bind with different subclasses of animals’ immunoglobulins G, although affinities for these proteins may vary from one animal species to another (9). In practice, protein A can be used efficiently against sera obtained from humans, donkeys, rabbits, dogs, pigs, and guinea pigs (7). SPA has also other biological activities such as Fab binding, activation of the complement system, hypersensitivity reactions, activation of inflammation through TNFR1 (Each of the repeated domains), cell-mediated cytotoxicity, interferon induction, activation of polyclonal antibody synthesis and mitogenic stimulation of lymphocytes (7,10,11). Immunological processes can benefit from SPA due to following characteristics; SPA has no effect on immunoreactivity of the antibody since the location of its binding site is situated in the Fc region, highly denatured SPA can be renatured and become functional, and the SPA-antibody linkages are reversible and can be broken by lowering the pH of the milieu (7). Accordingly, SPA is a useful immunological tool for the detection, isolation, and purification of immunoglobulins (immunochromatography). Consequently, many immunological assays such as ELISA (enzyme-linked immunosorbent assay), IFA (immunofluorescence assay), RIA (radio immune assay), Immunoblotting, Dot-ELISA can benefit from SPA.

SPA can be produced from large-scale cultures of wild type *S. aureus* strains by lysis of cell suspensions. Obviously, it is hard to cultivate the bacteria and extract this protein with high purity. Therefore, production of a recombinant type of this molecule can be very useful and yields more pure protein. Several commercial companies have begun to produce purified protein A that has become an expensive necessity for biological laboratories.

## 2. Objectives

The present study was conducted to construct an encoding plasmid vector for the expression of a recombinant fusion protein type of staphylococcal protein A in *E. coli*. This approach can be helpful in achieving higher amounts of recombinant SPA with ease of purification. The other objective of the study was to conjugate the SPA fusion protein to horseradish peroxidase enzyme, in order to evaluate its reactivity with human IgG molecules in immunological assays.

## 3. Materials and Methods

### 3.1. DNA Extraction and PCR

A strain of *Staphylococcus aureus* (ATCC 6538) was prepared from Iranian research organization for science and technology (IROST). DNA of the bacterium was extracted by a commercial DNA extraction kit (BioNEER, Korea) according to the manufacturer’s protocol. Considering selected plasmid vector for cloning, pMAL-c2X, and restriction enzymes, *EcoR* I and *Sal* I, primers for the coding region of protein A were designed using one of the present consensus sequences in GenBank (EU695225.1). The sequence of the forward primer was 5′-CTTGAATTCCAACACGACGAAGCT- 3′ and of the reverse primer was 5′-CGCGTCGACTTATGCATCATTTAGCT-3′ (recognition sites of the restriction enzymes *EcoR* I and *Sal* I are underlined). This pair of primers was designed to amplify 4 out of 5 domains of encoding DNA fragment of the SPA. Protein A coding region (687 bp) was amplified by PCR using following thermal cycling program; predenaturing at 94ºC for 3 min; 35 cycles of [denaturing at 94ºC for 1 min, annealing at 40ºC for 1 min, extension at 72ºC for 70 s], and final extension at 72ºC for 10 min.

### 3.2. Construction of the Recombinant Plasmid

The amplified fragment and the plasmid vector (pMAL-c2X) were digested with the restriction enzymes (Fermentas, Lithuania) and ligated at 22ºC for 70 min by T4 DNA ligase (Fermentas, Lithuania) followed by inactivation at 80ºC for 20 min. *Escherichia coli* (TG1 strain) was transformed with the plasmid according to (12). Bacterial clones containing the recombinant plasmid were cultured on ampicillin embedded LB agar. The plasmids were isolated and the presence of the desired fragment was examined by digestion assay according to the manufacturer’s protocol.

### 3.3. Expression of the Recombinant Maltose Binding Protein-protein A (MBP-SPA) Fusion Protein

Bacterial colons, which contained recombinant plasmids were cultured in LB broth enriched with 20 mM glucose until OD_600_ reached 0.5. Thereafter, IPTG (Isopropyl-β-D-thiogalactopyranoside) was added to the cultures followed by cultivation for another 4 h. Expression of the recombinant protein (~67 kDa) was examined by SDS-PAGE.

### 3.4. Purification of MBP-SPA Protein

IPTG-induced culture (0.5 l) of the selected recombinant bacterial colony was centrifuged at 6000 ×g for 10 min (4ºC) and the pellet was resuspended in column buffer (20 mM Tris-HCl, 200 mM NaCl, 1 mM EDTA) and sonicated. After centrifugation at 12000 ×g for 30 min (4ºC), the precipitate was discarded and the recombinant MBP-SPA protein was purified from the supernatant using an amylose-resin column chromatography according to the instruction manual of the pMAL protein purification system (New England Biolabs, USA). Finally, the purified recombinant protein was examined by SDS-PAGE.

### 3.5. Western Blot

After electrophoresis of the recombinant protein A and purified MBP (as a negative control) on 10% SDSpolyacrylamide gel, protein bands were transferred to nitrocellulose membrane using semi-dry procedure at 12 V for 2 h. Membrane was blocked in PBS contained 0.2% Tween 20 at 22ºC for 2 h and the membrane was rinsed three times with PBST (PBS with 0.05% v/v Tween 20). Immunoblot assay was carried out in one step procedure. The target recombinant protein was detected using 1 h incubation with 1/1000 dilution of a rabbit anti-chicken IgG conjugated to horseradish peroxidase (HRP) (Sigma, USA) at 22ºC. Conjugated rabbit anti-chicken IgG was diluted in PBST contained 1% Ovalbumin (Merck, Germany). After washing in PBST and PBS, the reaction was developed by immersion of the membrane in substrate-chromogen solution (H_2_O_2_ and 4-chloro-1-naphthol).

### 3.6. Conjugation of the Recombinant MBP-SPA Protein to Peroxidase Enzyme

The conjugation process was carried out based on a previously described procedure (7). Briefly, the purified recombinant protein (1 mg.mL^-1^) was dialysed at 4ºC for 24 h against 20 mM sodium carbonate buffer, pH 9.5 (two changes of buffer). On the other hand, 1 mg of a type II horseradish peroxidase enzyme (Sigma, USA) was dissolved into 1.2 mL of distilled water and mixed with 0.3 mL of sodium phosphate buffer containing 0.1 M sodium periodate and incubated at 22ºC for 20 min followed by dialysis in 1 mM sodium acetate buffer, pH 4 (two changes of buffer). Afterwards, MBP-SPA protein and peroxidase enzyme solutions were mixed together and incubated at 22ºC for 2 h. Sodium borohydride (0.1 mL of 0.1 M) was added to the mixture and incubation was continued at 4ºC for 2 h followed by dialysis in PBS for 24 h. Equal volume of glycerol and 20 mg.mL^-1^ of purified egg albumin (Sigma, USA) was added to the mixture and the prepared peroxidase conjugated MBP-SPA protein was stored at 4ºC.

### 3.7. Reactivity of the Conjugated MBP-SPA Protein with Human IgG in ELISA

A: Purified human IgG, diluted in carbonate/bicarbonate buffer, was applied at 1 μg.welL^-1^ into a 96-well polystyrene microtiter plate (Karizmehr, Iran) as ELISA antigen and incubated for 16 h at 4°C. A purified chicken IgG was also applied at the same concentration as negative control. All subsequent incubations were at 22ºC and the plates were washed three times with PBST after each step. Blocking of unreacted sites was carried out for 3 h using PBST containing 1% ovalbumin. Each dilution (1:100 to 1:25600) of the peroxidase conjugated MBP-SPA protein in PBST (50 μL) were added to the wells twice and incubated for 45 min followed by 3 consecutive washes with PBST.H_2_O_2_ (50 μL) and tetramethylbenzidine solution (substrate-chromogen) was added to each wells and the plate was incubated for 10 min at dark. Finally, 50 μL of stop solution (0.1 M HCl) was added to each well and optical density (OD) values were measured at 450 nm with a plate reader (Pishtazteb, Iran).

B: Purified human IgG dilutions were applied at 0.015 to 1 μg.welL^-1^ (0, 0.015, 0.031, 0.062, 0.125, 0.25, 0.5, 1 μg.welL^-1^) twice into a 96-well polystyrene microtiter plate as ELISA antigens and incubated for 16 h at 4ºC (purified chicken IgG dilutions were also applied with the same concentrations as negative controls). Blocking of unreacted sites was done as previously described and after washing the plate, 50 μL of 1:500 dilution of the peroxidase conjugated MBP-SPA protein in PBST was added to each well and the plate was incubated for 45 min followed by 3 washes with PBST. Further steps were the same as the previous ELISA.

## 4. Results

### 4.1. Expression and Purification of the Recombinant MBP-SPA Protein

**Figure 1:**
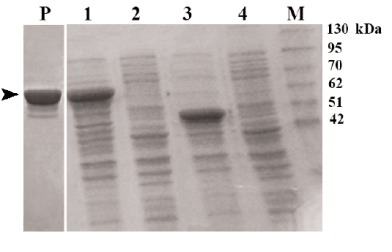
The electrophoretic pattern of the expressed recombinant protein A. Lane M represents a chromatein prestained protein ladder (Vivantis), Lanes 1 and 2 exhibit expression patterns of the expressed recombinant protein A fused to MBP (67 kDa) after and before adding IPTG. Lanes 3 and 4 represent expression patterns of whole MBP molecule after and before adding IPTG (intact MBP, ~50 kDa) by control bacterial cells contained pMAL-c2X without any target gene. Lane P depicts the purified recombinant protein A

Considering the maltose binding protein encoded by pMAL-c2X as a fusion partner at the beginning of the recombinant protein, predicted molecular weight of the whole protein was about 67 kDa (42 kDa for MBP + 25 kDa for recombinant protein A, (Figure 1). The purified recombinant protein A with amylose resin column chromatography was also shown in the Figure 1.

### 4.2. Western Blot

**Figure 2:**
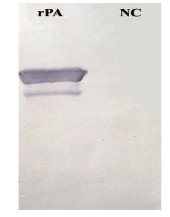
The result of the western blot assay. The right lane (NC) contained the purified maltose binding protein as the negative control. The left lane depicts the expressed recombinant protein A, purified by amylose resin. It also seems that protein A was somehow broken but all parts still had the ability to bind to the conjugated IgG molecules

The expressed recombinant MBP-SPA and MBP (as negative control) were subjected to western blotting. As shown in Figure 2, the expressed recombinant protein A was detected by applying a conjugated antibody and no detectable false reaction was noted in the lane for the negative control. Distinctly, this recombinant SPA is fully functional and can be used in various immunological assays.

### 4.3. Reactivity of the Peroxidase Conjugated MBPSPA Protein with Human IgG in ELISA

**Figure 3:**
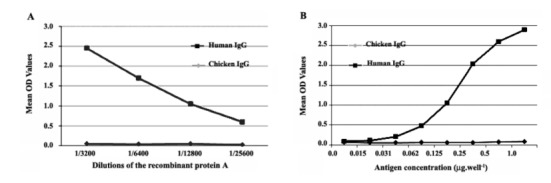
A: Reactivity of human IgG molecule (1μg.welL^-1^) with serial dilutions of the peroxidase conjugated recombinant protein A (chicken IgG was used as negative control). B: Reactivity of consecutive concentration of human IgG molecule with the 1:500 dilution of the peroxidase conjugated recombinant protein A

A: The OD values obtained from this ELISA showed that the reactivity of the peroxidase conjugated recombinant protein A, up to 1:1600 dilution of the protein, was out of the reading range of the plate reader. However, OD values of more diluted samples were measurable. Therefore, the mean OD values of the dilutions higher than 1:3200 are shown in Figure 3A. The peroxidase conjugated recombinant protein A was able to detect human IgG molecules even at 1:25600 dilution and did not show any reaction with the chicken IgG molecules (negative control).

B: Different concentrations of human IgG molecules were applied to determine the reactivity of these IgG molecules with a constant dilution (1:500) of the peroxidase-conjugated recombinant protein A. The OD values obtained from this ELISA exhibited that the peroxidase conjugated recombinant protein A has a good capacity for binding to human IgG molecules. Figure 3B depicts the mean OD values for this ELISA.

## 5. Discussion

The staphylococcal protein A is one of the valuable bacterial proteins that can be used in several immunological assays depending on immunoglobulin types and subclasses. It is not only used for detection of immunoglobulins, but also for isolation of IgGs through immuno-chromatography (13,14). However, it also binds to CRP (C-Reactive Protein), a clinically important acute phase protein and both glycosylated and non-glycosylated CRPs show calcium-independent differential-binding to this protein (15). Thus, applied antibodies (sera) must be considered if they have been taken from infected animals or animals with inflammation. Several studies have been conducted to find promising applications of this invaluable bacterial protein. Inouye and Sahara (2008) constructed a cold induced expression vector in *E. coli* cells that consists of a histidine tag sequence for nickel chelate affinity purification, IgG-binding domain of protein A, and the multiple cloning sites (16). Iijima *et al.* (2011) developed nanocapsules for displaying IgGs on immunosensor chips using IgG Fc-binding domain derived from SPA (17).

Furthermore, one of the major problems in serodiagnosis in wild animals is unavailability of specific antiglobulin conjugate molecules. In an interesting study, Bhide *et al.* (2004) focused on validation of protein A/G dependent ELISA in game animals like deer and mouflons as well as in hunting dogs. They showed that ELISA is a sensitive and promising diagnostic tool in serodiagnosis of Lyme disease in game ungulates and this protein can be used effectively for serosurvey in different wild mammals (18). It could be inferred that there are possibly other animal species immunoglobulins that SPA can bind to them. These characteristics of SPA make it inestimable protein for commercial companies to produce such a molecule and sell it with high price. As a result, construction of an accessible recombinant SPA by researchers in their own labs may help them to decrease their expenses and moreover, it will also be available anytime in the lab for further applications. This protein can also be modified to produce a protein with specific characteristics. When coupled to radioactive, enzymatic (e.g. alkaline phosphatase or horseradish peroxidase) or fluorescent tags, it is an excellent reagent to investigate antibodies with high affinity for protein A (7).

In the present study, the coding region of protein A gene was cloned into a prokaryotic plasmid vector, pMAL-c2X. High amount of the recombinant protein A fused to MBP was easily expressed by *E. coli* and purified using amylose resin column chromatography. The process of purification can also be cheaply performed (19). Moreover, MBP can enhance the solubility of the expressed recombinant proteins (20,21). The purified recombinant protein A was conjugated to horseradish peroxidase enzyme to construct a peroxidase recombinant protein A. This conjugated recombinant SPA showed high affinity for binding to human IgG molecules in the ELISAs as even 1:500 dilution of it was able to react with 0.5 μg.welL^-1^ of human IgG with the OD value approximately equal to 2.5. Producing such a recombinant molecule can be very useful in the laboratories and this functional recombinant SPA surmounts many of research requirements. For example, the recombinant SPA itself can be used for the purification of antibody molecules in antibody affinity chromatographies and a HRP-conjugated one may be used in various immunological assays such as ELISA, Dot-ELISA, western blot, and immunohistochemistry as an indicator anti-antibody molecule. Moreover, the recombinant protein A can also be conjugated with other molecules (e.g. biotin, fluorescent dyes, other enzymes, etc.) and this may broaden the application range of this molecule to be used in more research areas.

## Acknowledgements

This project was supported by research grants from Shahid Chamran University of Ahvaz, Ahvaz, Iran.
